# Long non-coding RNASNHG17 promotes the progression of breast cancer by sponging miR-124-3p

**DOI:** 10.1186/s12935-020-1129-y

**Published:** 2020-02-05

**Authors:** Ye Du, Na Wei, Jinghui Hong, Weiyun Pan

**Affiliations:** 1 Department of Breast Surgery, The First Hospital of Jilin University, Changchun, 130021 Jilin People’s Republic of China; 2 Department of First Operating Room, The First Hospital of Jilin University, Changchun, 130021 Jilin People’s Republic of China; 3 Department of ICU, The First Hospital of Jilin University, Changchun, 130021 Jilin People’s Republic of China

**Keywords:** Breast cancer, SNHG17, miR-124-3p

## Abstract

**Background:**

Small nucleolar RNA host gene 17 (SNHG17), a novel cancer-related long noncoding RNA (lncRNA), was reported to be responsible for processing and developing in several cancers. Nonetheless, the clinical significance and biological function of SNHG17 in human breast cancer (BC) remain rarely known.

**Materials and methods:**

58 pairs of BC tissues and adjacent non-cancerous tissues were harvested to measure SNHG17 expression levels. SNHG17 was knockdown to study its biological behavior in BC cells. The microRNAs (miRNAs) that can bind to SNHG17 were predicated using Starbase2.0 and were tested using luciferase reporter activity and RIP assays. A xenograft model was established to investigate the impact of SNHG17 in tumor growth in vivo.

**Results:**

An increased SNHG17 was observed in BC samples and cell lines compared with corresponding control. Increased SNHG17 was closely associated with poor prognosis.SNHG17 depletion suppressed cell proliferation, migration and invasion in vitro, as well as inhibited tumor growth in xenograft tumor models. Mechanistically, SNHG17 could function as an endogenous sponge of miR-124-3p in BC cells. Moreover, the repression of cell proliferation, migration and invasion induced by SNHG17 knockdown would reversed by miR-124-3p inhibitor.

**Conclusion:**

The present study demonstrated that the lncRNASNHG17 could regulate the progression of BC by sponging miR-124-3p.

## Background

Breast cancer (BC) is one of the most prevalent types of malignancy and a leading cause of cancer-related death among women worldwide [1]. Despite recent advances in surgery, chemotherapy and radiotherapy, the prognosis of BC remains poor because of its heterogeneous nature and metastasis to other organs [2, 3]. Therefore, there is an urgent need to explore molecule mechanism involved in BC progression to develop new therapeutic targets for this cancer.

In recent years, non-coding RNAs (ncRNAs) have gained widely attention as studies have shown that they are involved in various critical pathophysiological processes, including cell proliferation, metastasis and invasion, and so on [4, 5]. The ncRNAs includes long noncoding RNAs (lncRNAs, a type of ncRNAs over 200 nucleotides in length and with limited protein-coding ability) [6], and microRNAs that inhibit target gene expression by binding to the 3′ UTR (3′ untranslated region) of a target gene [7]. Both lncRNAs and miRNAs were shown to be involved in the regulation of the initiation and development of various cancers including BC [8, 9]. A body of evidence demonstrated that lncRNAs could serve as competing endogenous RNAs (ceRNAs) for miRNAs to regulate miRNAs and miRNA-targeted mRNAs [10].

Small nucleolar RNA Host Gene 17 (SNHG17), an important lncRNA, was proven to play oncogenic role in non-small cell lung cancer [11], gastric cancer [12], colorectal cancer [13] and melanoma [14]. However, the biological function of SNHG17 in the initiation and development of BC remains poorly understood. Therefore, we strive to elucidate the functional roles of SNHG17 in BC. We found that knockdown of SNHG17 inhibited the growth of BC in vivo and in vitro. We also selected miRNAs that bind to SNHG17 using starbase2.0, and identified that miR-124-3p, a known tumor-suppressive miRNA in BC [15–18], was a direct target of miR-124-3p. We investigate whether the SHNG17 regulate the progression of BC by sponging miR-124-3p.

## Materials and methods

### Clinical tissue specimens

A total of 58 pairs of BC tissues and paired non-tumor tissues were collected from the First Hospital of Jilin University from Jan 2013 to Jan 2014. All the experiments concerning the use of patient samples in this stud were conducted in accordance with the Declaration of Helsinki and were approved by the Medical Ethics Committee of Jilin University. Written informed consents were acquired from all patients. All tissues were stored at in liquid nitrogen. The clinical features of the patients with BC were listed in Table 1.

**Table 1:** Association of SNHG17 expression with clinicopathologic factors of 58 patients with BC

Variables	No. of cases	SNHG17 expression		*P* value
		High	Low	
Age (years)				*P *= 0.5936
< 50	22	11	11	
≥ 50	36	21	15	
ER				*P *= 0.1103
Positive	34	22	12	
Negative	24	10	14	
TNM stage				*P *= 0.0014
I–II	44	19	25	
III–IV	14	13	1	
Tumor size				*P *= 0.2685
≤ 2 cm	40	20	20	
> 2 cm	18	12	6	
Lymph node metastasis				*P *= 0.0249
No	45	21	24	
Yes	13	11	2	

### Cell lines and culture

Human normal human breast epithelial cell (MCF-10A) and four breast cancer cell lines (MCF-7, MDA-MB-231, MDA-MB-468 and BT-474) were purchased from the ATCC (Manassas, VA, USA). L-15 medium (Gibco, CA, USA) supplemented with 10% FBS (fetal bovine serum, Gibco) was used to culture the cells in a high humidity incubator (37 °C; 5% CO_2_).

### Virus infection and cell transfection

Recombinant lentiviruses expressing the sh-SNHG17 (shRNA directly targeting SNHG17; 5′-GAUUGUCAGCUGACCUCUGUCCUGU-3′) and sh-NC (negative control shRNA; 5′-UUCUCCGUUCGUGUCACGUUU-3′) were bought from Sangon Biotech Company (Shanghai, China). MCF-7 and MDA-MB-231 were transfected with sh-NC or sh-SNHG17 lentiviral transduction particles (MOI = 20) using 5 μg/mL polybrene (Genechem). Stably transfected cells was chosen with 1 μg/ml puromycin (Calbiochem, USA) for 2 weeks. The mimics and inhibitor of miR-124-3p and their controls (miR-NC) were bought by GenePharma (Shanghai, China), and were transfected with MCF-7 and MDA-MB-231 cells using Lipofectamine 2000 (Invitrogen) in 6-well plates when cells grown to 70% confluent.

### Quantitative real-time polymerase chain reaction (qR-TPCR)

Trizol reagent kit (Invitrogen) was applied to isolate total RNA from cultured cells or tissues. The concentration and purify of RNA was determined using Nano-Drop 2000 spectrophotometer (Termo Scientific, USA). The PrimeScript™ RT reagent Kit (Takara, Japan) was used to synthesis cDNA. The expression of miR-124-3p was determined using miRNA qRT-PCR Starter kit (Riobobio, USA) using primers of U6 and miR-124-3p (Riobobio). SYBR^®^ Green Real time PCR Msater Mix (Toyobo, Japan) was used to detect SNHG17 expression. Expression levels of miR-124-3p and SNHG17 were normalized by U6 and GADPH, respectively. The relative expression of miR-124-3p and SNHG17 was calculated by using 2^− ΔΔCt^ method.

### RNA isolation of nuclear and cytoplasmic fractions

The NE-PER™ Nuclear and Cytoplasmic Extraction Reagents Kit (Thermo Scientific, USA) was used to isolate and collect cytosolic and nuclear fractions according to manufacturer’s protocol. The expression levels of SNHG17, U6 (nuclear control transcript) and GAPDH (cytoplasmic control transcript) were examined in cytoplasmic and nuclear fractions using qRT-PCR as described above-mentioned.

### Cell proliferation assay

Cell Counting Kit-8 (CCK-8; Dojindo, Japan) and colony formation assays were used to determine cell proliferation. In CCK-8 assay, stable transfected cells were collected and seeded into 96-well plates at a den-sity of 1 × 10^4^ cells per well. After 24, 48 or 72 h (hs), cells was treated using 10 μl CCK-8 assay for 2 h. The proliferation ability was determined by measuring absorbance at 450 nm using an enzyme immunoassay analyzer (Biorad, Hercules, CA, USA).

For colony formation assay, 1000 stable transfected cells were added into six-well plates and cultured for 10 days. After washed with PBS, colonies were fixed with methanol and stained with 0.1% crystal violet (Sigma-Aldrich, StLouis, MO, USA) for 15 min. The visible colonies were counted.

### Cell migration and invasion assays

Wound healing assay used to assess cell migration ability as described previously [19]. Stable transfected cells were seeded in six-well plates until full confluence. The monolayer cells were manually scraped to create wound area using a 100 µl sterile pipette tip. The wounding area at 0 h and 24 h was measured and imaged a light micro-scope (Olympus, Tokyo, Japan).

Transwell invasion assay was performed to assess cell invasion. Briefly, stably transfected cells were suspended in serum-free medium and added to upper transwell chambers (BD Biosciences, Sparks, MD, USA) pre-coated with BD BioCoatMatrigel. After incubation 24 h, invasive cells were fixed with methanol, stained with 0.1% crystal violet, and counted at five randomly selected fields in each well using a light micro-scope (Olympus).

### Luciferase reporter assay

Oligonucleotides that encode SNHG17 cDNA encompassing miR-124-3p-binding sites were synthesized and inserted into pGL3 vector (WT-SNHG17).We also constructed a pGL3-Report plasmid that carried the mutant SNHG17 binding region (MUT-SNHG17). For the luciferase reporter assay, MCF-7 and MDA-MB-231cells were co-transfected with luciferase reporter plasmids and negative control (miR-NC) or miR-124-3p mimics using Lipofectamine 2000. A double-luciferase assay system (Beyotime, Wuhan, China) was used to determine the luciferase activity at 24 h post-transfection.

### RNA-binding protein immunoprecipitation (RIP) assay

MCF-7 and MDA-MB-231 cells transfected with miR-124-3p mimics or miR-NC were harvested and subjected to RIP assay using an anti-Ago2 antibody and a RIP kit (Millipore Inc., Bedford, MA, USA) according to the manufacturer’s instructions. Mouse anti-human immunoglobulinG (IgG) antibody was used as control. Finally, RNA was extracted and quantified by qRT-PCR to examine the expression of SNHG17 as described above-mentioned.

### Animal experiment

Female athymic BALB/c nude mice (5 weeks old, 18–20 g) were bought from Laboratory Animal center of Jilin University. All of the animal experiments were performed based on experimental protocols and approved by the Institutional Animal Care and Use Committee of the Jilin University.

To initiate tumor xenografts, 2 × 10^6^ MCF-7 cells that were stably transfected with sh-NC or sh-SNHG17 were subcutaneously injected into the flank of mice. Tumor growth was measured by caliper measurements every week and was calculated based on following formula: volume = 0.5 × length × width × width. After 5 weeks, all mice were euthanized by intraperitoneal injection of 200 mg/kg pentobarbital. Tumor tissues were removed and weighed. A part of tumor was used for immunohistochemical staining to detect the expression of Ki-67 (a proliferation marker) as described previously [20].

### Statistical analysis

All results are presented as the mean ± SD of at least three independent experiments, are analyzed using the SPSS20.0 statistical software package. Student’s t-tests and one-way ANOVA were used to determine statistically significant differences between groups. The Chi-square test was used to analyze the correlation between the expression of SNHG17 and clinical parameters of patients with BC. Kaplan–Meier method and a log-rank test were used to assess overall survival rate. Spearman’s correlation coefficient was used to assess correlation between two groups. A *P* value < 0.05 was considered statistically significant.

## Results

### Upregulation of SNHG17 is associated with poor prognosis of BC patients

**Fig. 1:**
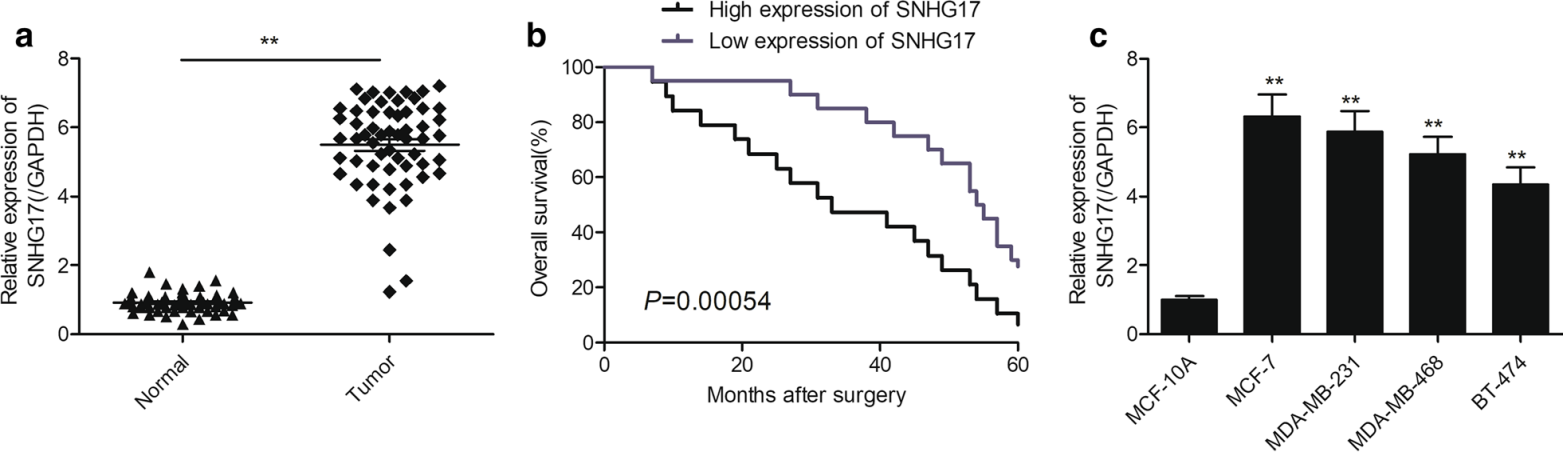
SNHG17 is upregualted in BC tissues and correlated with poor outcomes in patients with BC. **a** Relative expression of SNHG17 in 58 BC tissues and corresponding adjacent normal breast tissues. **b** Kaplan–Meier overall survival curves based on SNHG17 expression levels. **c** SNHG17 expression in human normal human breast epithelial cell (MCF-10A) and four breast cancer cell lines. *P < 0.05, **P < 0.01

To evaluate the expression pattern of SNHG17 in BC, we first examined the expression of SNHG17 in the BC tissues and adjacent normal tissues. As shown in Fig. 1a, the expression of SNHG17 was upregulated in BC tissues compared with non-tumor breast tissues. To assess the association with clinical characteristic of BC patients and SNHG17 expression, we divided the patients to high-expression group (n = 32) and low-expression group (n = 26) based on the median level of SNHG17 expression. Table 1 displayed that increasedSNHG17 expression was positively associated with advanced TNM stages (III–IV stages) and lymph node metastasis. Kaplan–Meier analyses revealed that high SNHG17 expression group has poorer survival than in low SNHG17 expression group (Fig. 1b). In addition, we found that SNHG17 expression was increased in four BC cell lines compared to MCF-10A cells (Fig. 1c).

### SNHG17 knockdown inhibits tumor growth of BC in vitro and in vivo

**Fig. 2:**
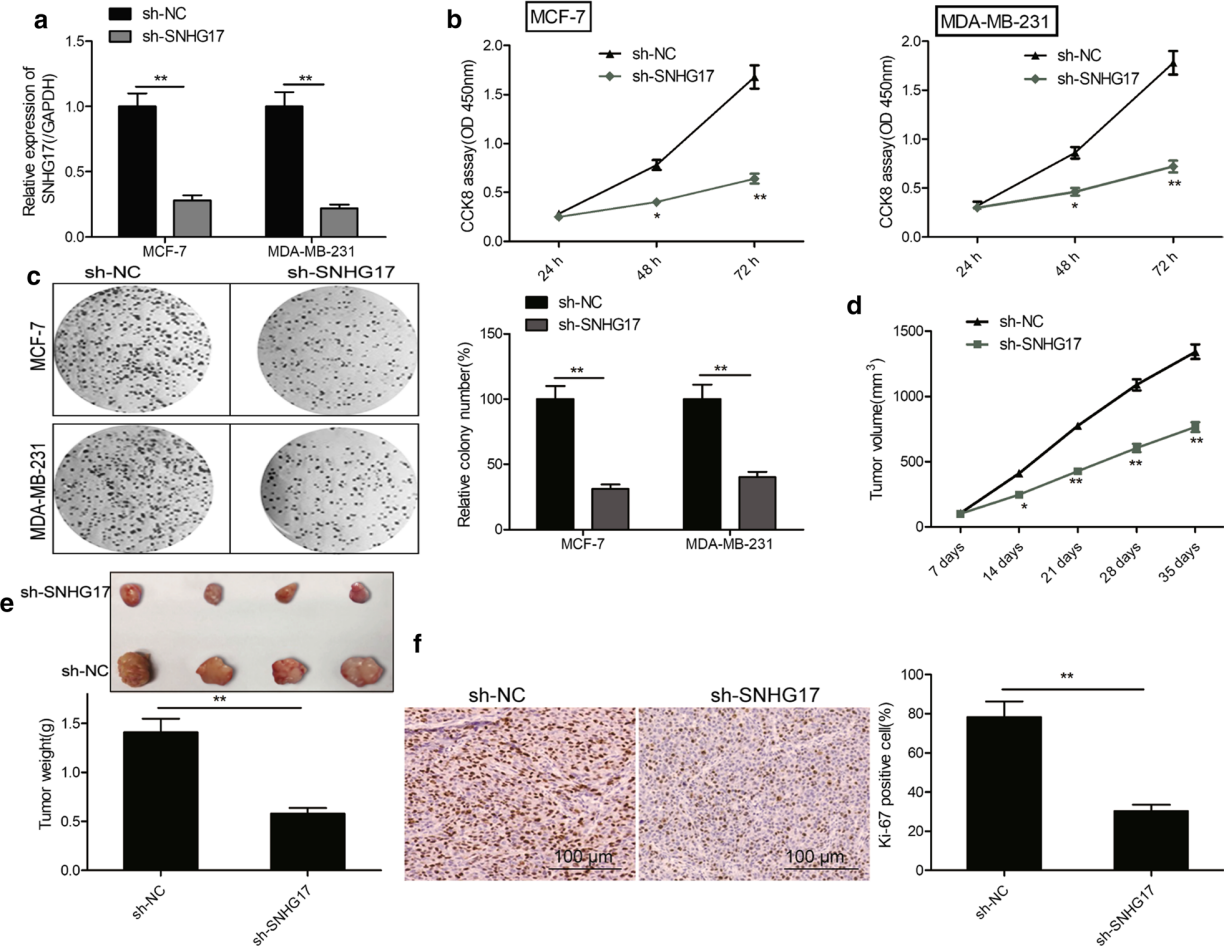
SNHG17 knockdown inhibits tumor growth of BC in vitro and in vivo. **a** The expression of SNHG17 in MCF-7 and MDA-MB-231 cells transfected with sh-NC or sh-SNHG17. **b** Effect of SNHG17 knockdown cell proliferation in MCF-7 and MDA-MB-231 cells. **c** Effect of SNHG17 knockdown on the colony formation of MCF-7 and MDA-MB-231 cells. **d** Tumor growth curves. **e** Tumor imaged and weight in xenograft tissues. **f** Ki-67 expression in xenograft tissues. **g** The expression of SNHG17 in xenograft tissues. *P < 0.05, **P < 0.01

To investigate the role of SNHG17 in BC cells, loss-of function experiments were performed in MCF-7 and MDA-MB-231 cells by transfection with shRNA against SNHG17 plasmid (sh-SNHG17). We found that transfection of sh-SNHG17 was able to decrease SNHG17 expression in MCF-7 and MDA-MB-231 cells (Fig. 2a). CCK8 assay revealed that knockdown of SNHG17 could result in a significant decrease of cell proliferation in MCF-7 and MDA-MB-231 cells (Fig. 2b). Consistent with this result, silence of SNHG17 inhibited colony formation of MCF-7 and MDA-MB-231 cells (Fig. 2c).

To investigate whether SNHG17 depletion affect the tumor growth in vivo, the xenograft tumor were established. We found that xenografts grew more slowly in SNHG17 depletion group than in sh-NC group (Fig. 2d). Moreover, the weight and size of tumors was decreased in the SNHG17 depletion group compared with those in the sh-NC group (Fig. 2e). More importantly, the Ki-67 expression was reduced in SNHG17 depletion group compared with sh-NC group (Fig. 2f). These results suggested that SNHG17 depletion inhibited BC growth in vitro and in vivo.

### SNHG17 knockdown inhibits migration and invasion of BC cells

**Fig. 3:**
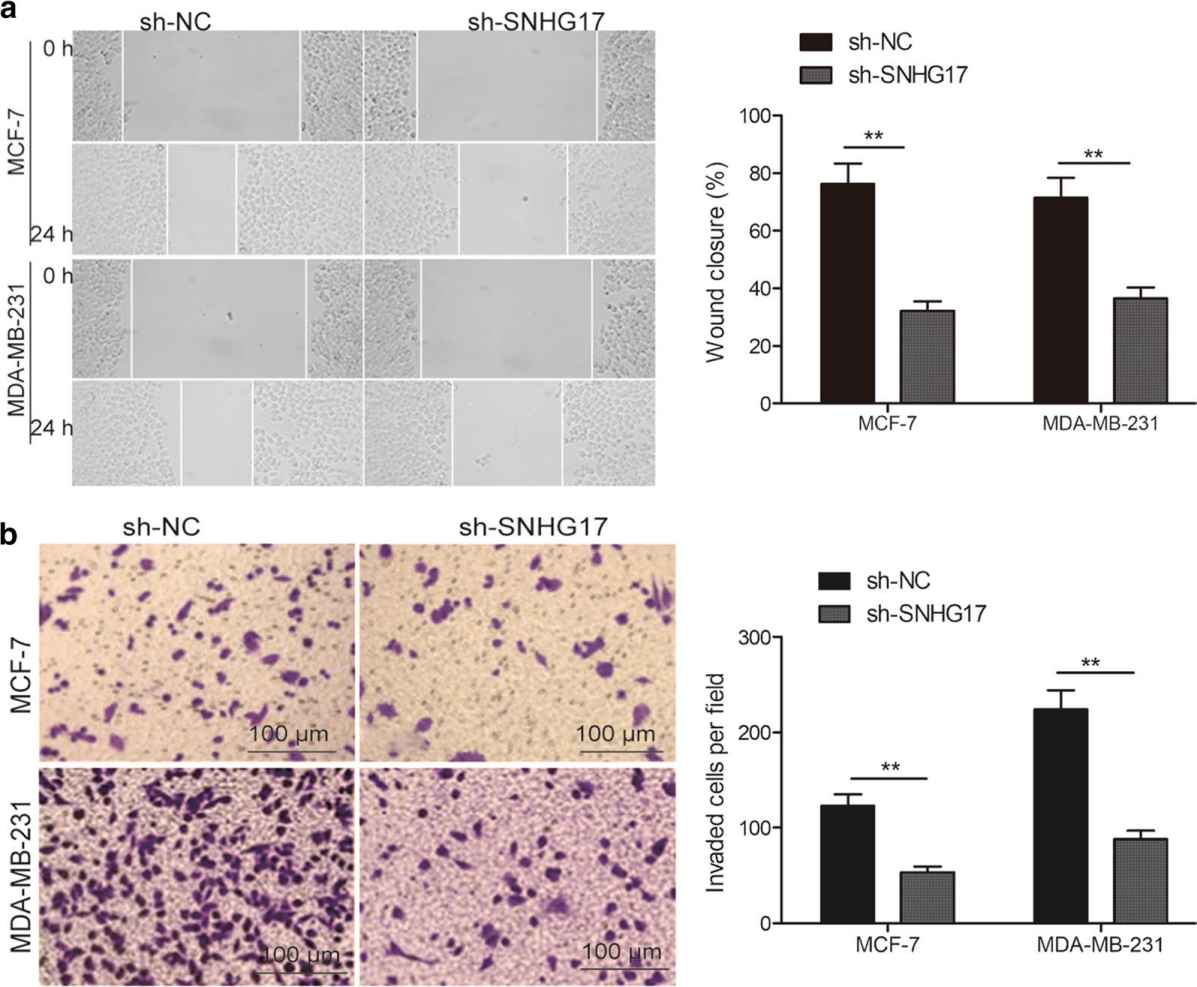
SNHG17 knockdown inhibits migration and invasion of BC cells. **a** The migration in MCF-7 and MDA-MB-231 cells transfected with sh-NC or sh-SNHG17. **b** The invasion in MCF-7 and MDA-MB-231 cells transfected with sh-NC or sh-SNHG17. *P < 0.05, **P < 0.01

Next, we checked the effects of SNHG17 knockdown on cell invasion and migration using the transwell assay and wound healing assay, respectively. The migration and migration capabilities were significantly decreased in MCF-7 and MDA-MB-231 cells transfection with sh-SNHG17 compared with cells transfected with sh-NC (Fig. 3a, b).

### miR-124-3p is a target of SNHG17 in BC cells

**Fig. 4:**
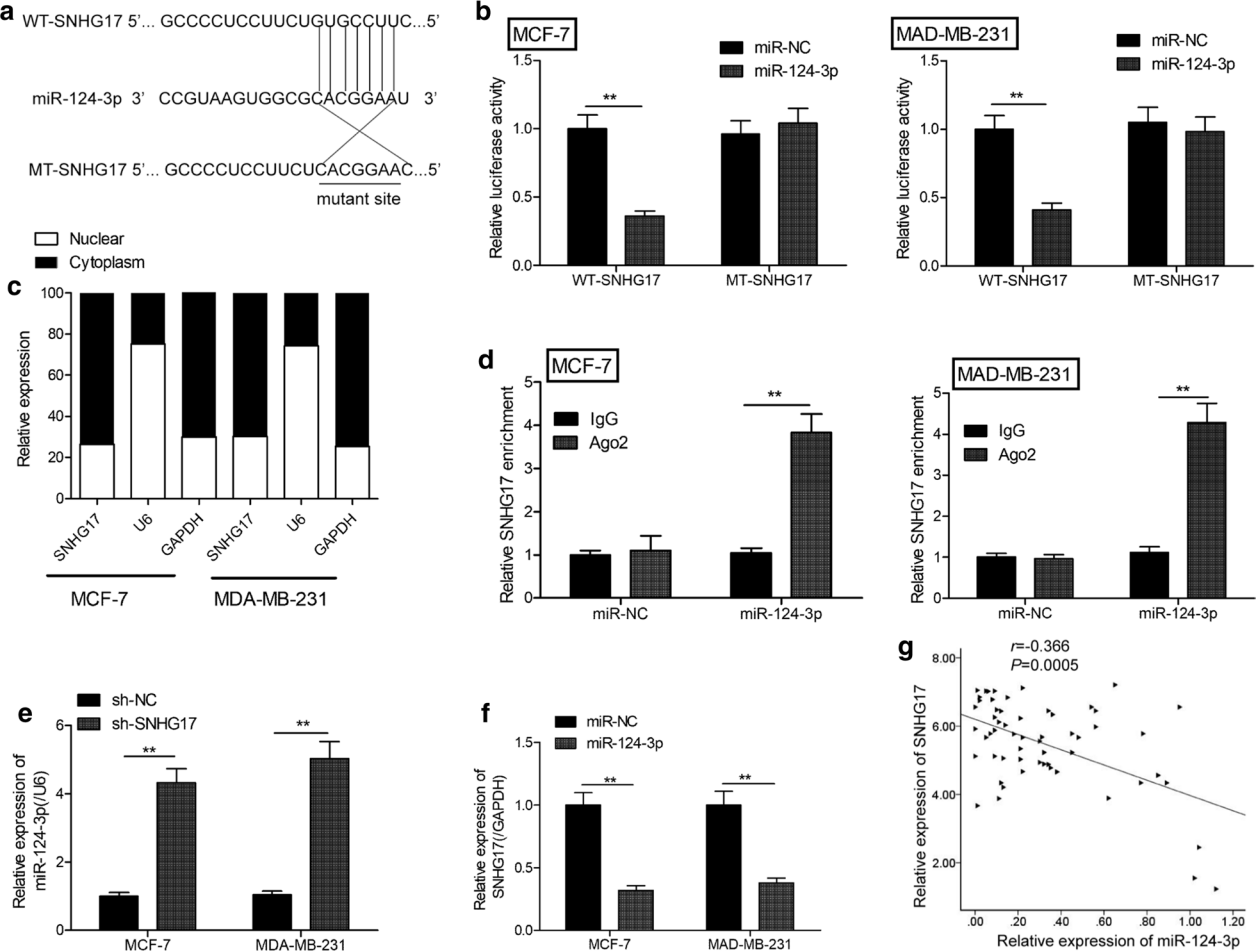
miR-124-3p is a target of SNHG17 in BC cells. **a** The predicted binding sites of miR-124 on the sequence of SNHG17 (WT-SNHG17). The target sequences of the SNHG17 were mutated (MT-SNHG17). **b** Luciferase activity in MCF-7 and MDA-MB-231cells co-transfected with miR-124-3p mimics or miR-NC, and luciferase reporter vector containing WT-SNHG17 or MT-SNHG17. *WT* wild-type, *MT* mutant-type. **c** The expression of SNHG17 in nuclear and cytoplasmic of MCF-7 and MDA-MB-231 cells by qRT-PCR. **d** The interaction between miR-124-3p and SNHG17 in MCF-7 and MDA-MB-231 cells were tested by RIP experiment. **e** The expression of miR-124-3p in MCF-7 and MDA-MB-231 cells transfected with sh-NC or sh-SNHG17. **f** The expression of SNHG17 in MCF-7 and MDA-MB-231 cells transfected with miR-NC or miR-124-3p mimics. **g** Spearman’s correlation coefficient analysis between miR-124-3p expression and SNHG17 expression in 58 patients with BC. *P < 0.05, **P < 0.01

To elucidate whether SNHG17 functioned as a ceRNA in regulating BC progression, Starbse2.0 software (http://starbase.sysu.edu.cn/starbase2/index.php) was used to predict potential target miRNAs of SNHG17. Among the candidates, miR-124-3p, a tumor suppressor miRNA that is frequently observed to be downregulated in BC [15–18], was selected and further study. As illustrated in Fig. 4a, there is a predicted targeting site of miR-124-3p on SNHG17. To test this predication, luciferase activity was performed. Our result demonstrated that miR-124-3p mimics could inhibit the luciferase activity in WT-SNHG17 group, with no effect in MT-SNHG17 group in MCF-7 and MDA-MB-231 cells (Fig. 4b). Moreover,we ascertain the subcellular localization of SNHG17 in MCF-7 and MDA-MB-231 cells. As illustrated in Fig. 4c, SNHG17 was mainly expressed in the cytoplasm of MCF-7 and MDA-MB-231 cells.RIP assays further demonstrated that miR-124-3p and SNHG17 were all enriched in Ago2-immunoprecipitation (Ago2-IP) relative to the control in MCF-7 and MDA-MB-231 cells (Fig. 4d). In addition, we found that knockdown of SNHG17 significantly increased miR-124-3p expression in MCF-7 and MDA-MB-231 cells (Fig. 4e), while overexpression of miR-124-3p led to a significant decrease in both MCF-7 and MDA-MB-231 cells (Fig. 4f). We also found that SNHG17 levels were negatively correlated with miR-124-3p levels in BC tissues (Fig. 4g). These results suggested that miR-124-3p bound to the transcript position of SNGH17.

### SNHG17 knockdown inhibits the progression of BC cells by regulating miR-124-3p

**Fig. 5:**
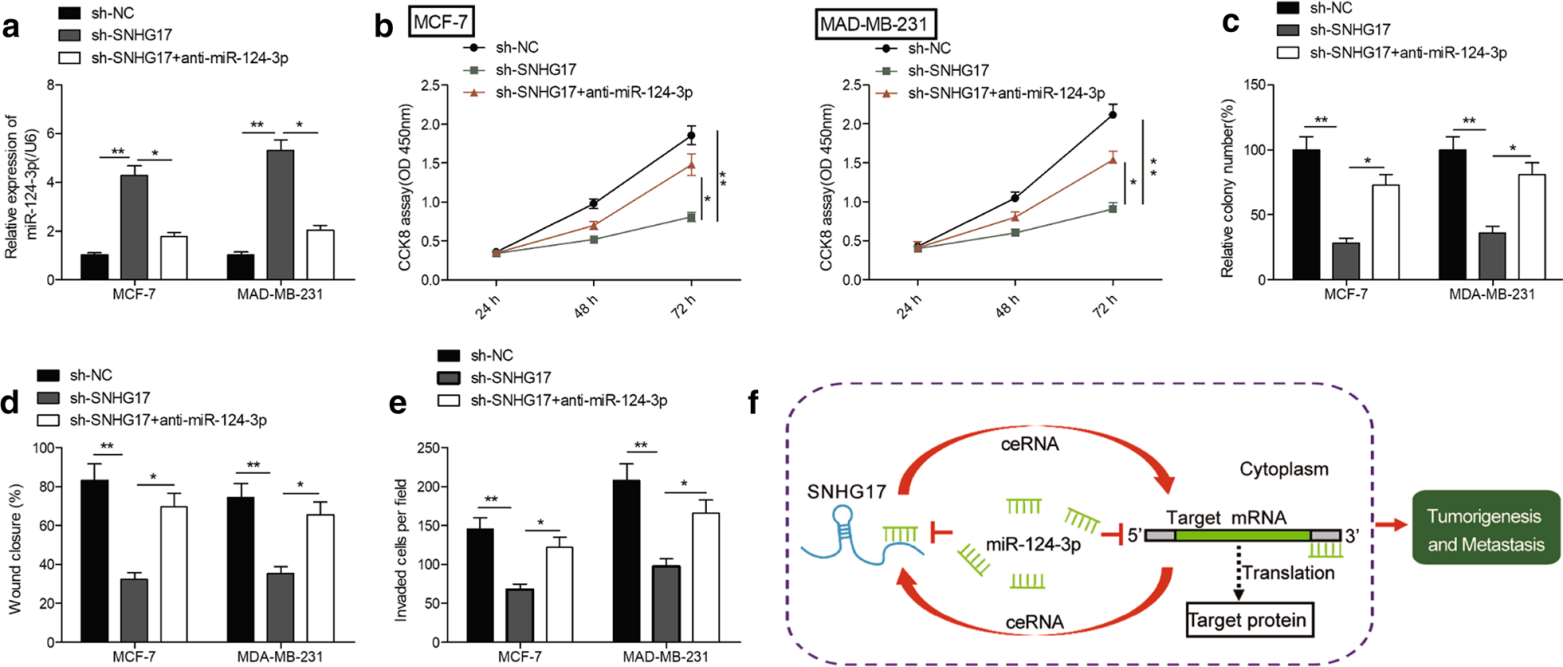
SNHG17 knockdown inhibits the progression of BC cells by regulating miR-124-3p. **a** The expression of miR-124-3p in MCF-7 and MDA-MB-231 cells transfected with sh-NC, sh-SNHG17 and sh-SNHG17 + miR-124-3p inhibitor(anti-miR-124-3p). **b–e** Cell proliferation, colony formation, migration and invasion in MCF-7 and MDA-MB-231 cells transfected with sh-NC, sh-SNHG17 and sh-SNHG17 + anti-miR-124-3p. **f** The schematic diagram of the mechanism of SNHG17/miR-124-3p axis in breast cancer. *P < 0.05, **P < 0.01

Considering the close correlation between miR-124-3p and SNHG17, we next evaluated whether the miR-124-3p expression implicates in biological effects by SNHG17 in BC cells. To this end, MCF-7 and MDA-MB-231 were transfected with sh-NC, sh-SNHG17 and sh-SNHG1 + miR-124-3p inhibitor. We found that transfection with sh-SNHG17 obviously increased miR-124-3p expression in MCF-7 and MDA-MB-231 cells, while transfection of miR-124-3p inhibitor partially reversed this trend (Fig. 5a). In addition,miR-124-3p inhibitor partially reversed the inhibitory effect on cell proliferation, colony formation, invasion and migration caused by SNHG17 knockdown in BC cells (Fig. 5b–e). In summary, these findings suggested that SNHG17 promoted BC growth and metastasis via modulation of miR-124-3p (Fig. 5f).

## Discussion

Multiple lncRNAs have been identified to serve as crucial modulators in modulating the progression of various cancers including BC [21, 22]. Zhu et al. revealed that lncRNA linc00460 drove BC progression via regulating the miR-489-5p/FGF7/AKT axis [23]. Yang et al. reported that lncRNAADPGK-AS1 promotes BC cell proliferation, migration, and epithelial-mesenchymal transition (EMT) process through regulating miR-3196/OTX1 axis [24]. Sun et al. demonstrated that SNHG7 promoted proliferation, invasion and EMT initiation of BC by sponging miR-34a and regulating Notch-1 pathway [25]. Here, we indicated lncRNA SNHG17 was increased expression in BC cell lines and tissues, and was closely associated with lymph node metastasis, advanced TNM stage, and poor overall survival ratio. Additionally, knockdown of SNHG17 inhibited BC cell proliferation, invasion and migration by regulating miR-124-3p.

Aberrant expression of SNHG17 has been reported to play oncogenic role in several cancers by regulating cancer cell proliferation, migration and metastasis [11–13, 26]. However, the role of SNHG17 in BC is not well known. Here, for the first time, we investigate the expression of SNHG17 expression in BC tissues and cell lines, and found that SNHG17 expression was upregualted and was associated with poor prognosis. Functional assay showed that downregulation of SNHG17 significantly inhibited cell proliferation and migration in vitro, and suppressed tumor growth in vivo. These results implied that SNHG17 plays an oncogenic role in BC progression.

Accumulating evidence illuminated that lncRNAs exerted biological various roles in various cancer by functioning as ceRNAs for sponging miRNA to regulating miRNAs expression [27]. Here, we found that SNHG17 expression was mainly enriched in the cytoplasm fraction of BC cells, indicating SNHG17 could interact with miRNAs. Through bioinformatics analysis, luciferase activity and RIP assays, we determined that miR-124-3p is a sponge of SNHG17 in BC cells.miR-124-3p has been to exhibit a low expression level and play a tumor suppressive role in BC by regulating multiple genes [15–18].Here, our result showed that miR-124-3p expression was negative correlated with SNHG17 expression in BC tissues.Based on rescue assays, we confirmed that miR-124-3p involved in SNHG17-mediated BC progression. These results implied that SNHG17 exerted an oncogenic role in BC partially by regulating miR-124-3p. However, miR-124-3p could regulate multiple targets genes, we would investigate whether SNHG17 could modulate these target genes in BC to further explore molecular mechanism of SNHG17 in this cancer.

## Conclusion

In conclusion, through integrating clinical data, we for the first time reveal that the SNHG17 is upregulated in BC tissues and is associated with poor prognosis. Through functional experiments, we showed that SNHG17 serve as an oncogene in BC facilitating cell proliferation, invasion and migration in vitro, as well as affecting tumor growth in vivo by sponging miR-124-3p, highlighting a clinical potential of SNHG17 as a novel biomarker and potential therapeutic target for BC.

## Data Availability

The data sets used and/or analyzed during the current study are available from the corresponding author upon reasonable request.

## Abbreviations

BC: Breast cancer
SNHG17: Small nucleolar RNA host gene 17
cDNA: Complementary DNA
CCK-8: Cell counting kit-8
IHC: Immunohistochemistry
RIP: RNA-binding protein immunoprecipitation (RIP)
WT: Wile type
MT: Mutant type
